# Synthesis and charge transfer characteristics of a ruthenium–acetylide complex†

**DOI:** 10.1039/d0ra08390a

**Published:** 2020-11-27

**Authors:** Robert Kuhrt, Po-Yuen Ho, Martin Hantusch, Franziska Lissel, Olivier Blacque, Martin Knupfer, Bernd Büchner

**Affiliations:** Leibniz-Institut für Festkörper- und Werkstoffforschung Dresden Helmholtzstr. 20 01069 Dresden Germany r.kuhrt@ifw-dresden.de; Leibniz-Institut für Polymerforschung Dresden Hohe Str. 6 01069 Dresden Germany; Department of Chemistry, University of Zurich Winterthurerstrasse 190 8057 Zürich Switzerland

## Abstract

A novel ruthenium–acetylide complex was synthesised and characterised in solid state and solution. Thin films of the complex were evaporated on silver and gold foils in ultra high vacuum in order to probe the electronic properties with photoemission spectroscopy. The charge transfer characteristics of the complex with the strong acceptor F_6_TCNNQ were investigated by UV-vis absorption in solution as well as at an interface with photoemission spectroscopy. A new excitation in the former optical gap of the pristine materials was probed in solution. Moreover, it was possible to identify the oxidised complex as well as the reduced acceptor by X-ray photoemission spectroscopy. In particular, our data reveal that oxidation of the complex mainly occurs at the Ru centre. The charge transfer can be characterised as localised and mainly ionic although signs of a reaction of the acceptors aminogroups with the ruthenium–acetylide complex were found.

## Abstract 2

A newly synthesised ruthenium–acetylide complex is characterised by various methods and its charge transfer to the acceptor F_6_TCNNQ is studied by optical and photoelectron spectroscopy.

## Graphical abstract

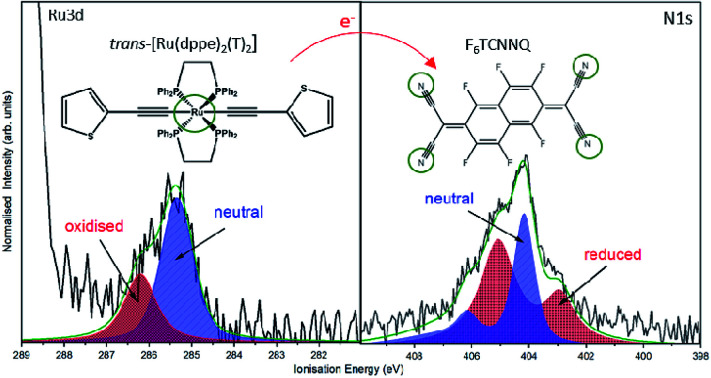

## 1 Introduction

Ruthenium–acetylide complexes were initially developed for the field of base-metal catalysis. More recently, their charge transfer behaviour was investigated by various methods, including mechanically controllable break junction (MCBJ), where this class of complexes demonstrated high conductivity^1,2^ and spectro-electrochemical analysis, in which their redox responsiveness and different charge transfer modes such as metal-to-ligand charge-transfer (MLCT), ligand-to-metal charge-transfer (LMCT) and even inter valence charge transfer (IVCT) were studied.^3–5^ Yet while the through-bond charge transfer behaviours were investigated by the above-mentioned methods, their through-space (or inter-molecular) charge transfer ability still remains ambiguous despite of its importance for organic electronic applications. Furthermore, unlike other Ru-compounds, which have been used as dopants for (organic) semiconductors, the doping ability of ruthenium–acetylide complexes is still unclear. In this study, we seek to fill this gap, and study the inter-molecular charge transfer ability of ruthenium–acetylide complexes. An exemplary complex, *trans*-[Ru(dppe)_2_(T)_2_], was synthesized, which can be understood as the repeating unit of a redox-active semiconducting polymetallayne.^6^ The acetylenes are end-capped with thiophene in order to yield a stable acetylide structure. The electron-rich character of the ruthenium moiety is utilized^7^ and charge transfer to an electron acceptor is observed and studied by photoemission spectroscopy. A charge transfer interface was formed with the strong electron acceptor F_6_TCNNQ, which was studied recently in combination with various other organic semiconductors.^8,9^

## 2 Synthesis and characterisation

**Fig. 1:**
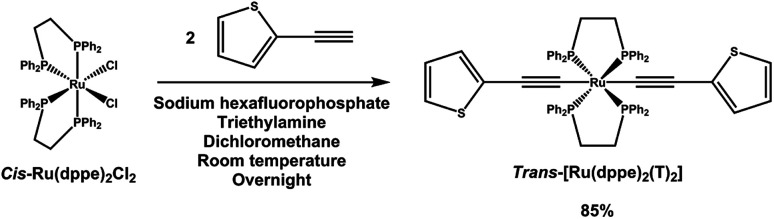
Synthetic route to *trans*-[Ru(dppe)_2_(T)_2_].

*trans*-[Ru(dppe)_2_(T)_2_] was synthesized *via* a dehydrohalogenation reaction starting from *cis*-Ru(dppe)_2_Cl_2_ (Fig. 1). *cis*-Ru(dppe)_2_Cl_2_ (0.50 g, 0.52 mmol) was added into a 100 mL round-bottom flask with 50 mL of dichloromethane (DCM). Sodium hexafluorophosphate (0.29 g, 1.7 mmol) was then added into the same flask. Once the hexafluorophosphate was added, the colour of the solution changed from bright yellow to deep red immediately. 2-Ethynylthiophene (0.18 g, 1.62 mmol) and trimethylamine (0.32 g, 3.13 mmol) were then added into the mixture. The colour of the mixture turned from red to yellow. The reaction mixture was stirred at room temperature overnight, then acetonitrile (ACN) was added and the DCM was removed on rotary evaporator causing a precipitate to form. The resulting precipitate was washed with ACN and then with hexane. The target compound was afforded as a yellow powder (0.49 g, 0.44 mmol, yield = 84.9%). Crystals suitable for X-ray analysis were grown from DCM.

The compound was fully characterized using ^1^H, ^13^C and ^31^P NMR, UV-vis in solution and thin film, Raman, electrospray ionization mass spectrometry (ESI-MS), elemental analysis and cyclic voltammetry. Furthermore the structure was confirmed by a single crystal X-ray diffraction experiment. Detailed information on the experimental results can be found in the ESI,† including the corresponding figures.

As expected for a centrosymmetric diamagnetic compound, the ^3^1P NMR spectra shows a singlet at 52.9 ppm, corresponding to the four chemically equivalent phosphorous atoms of the bidentate ligands. The symmetric vibration of the acetylide is detected in the Raman spectrum at 2064 cm^−1^. The cyclic voltammogram exhibits one fully reversible oxidation event at −0.073 V *vs.* the ferrocene/ferrocenium redox pair. The oxidation is metal-centred and corresponds to the Ru(ii)/Ru(iii) transition.

**Fig. 2:**
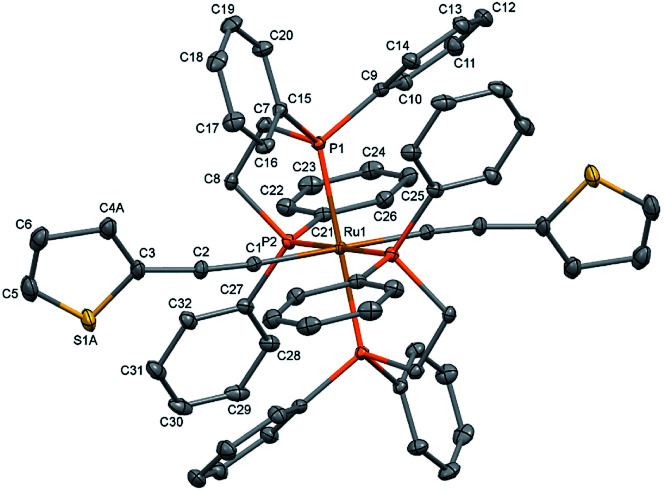
Molecular structure of *trans*-[Ru(dppe)_2_(T)_2_] (all hydrogen atoms are omitted for clarity). The thermal ellipsoids are drawn at the 20% probability level.

The single crystal X-ray diffraction data were collected at 160 K on a Rigaku Oxford Diffraction Synergy (Pilatus 200K detector) diffractometer equipped with an Oxford liquid-nitrogen Cryostream cooler and using the Cu Kα radiation (*γ* = 1.54184 Å). The pre-experiment, data collection, data reduction and analytical absorption correction^10^ were carried out with the program suite *CrysAlisPro* (*version 1.171.40.39a*).^11^ Using *Olex2*,^12^ the structure was solved with the SHELXT^13^ small molecule structure solution program and refined with the *SHELXL* program package^14^ by full-matrix least-squares minimization on F2. In the crystal structure (Fig. 2), the ruthenium centre lies on a centre of inversion, only half of the molecule was refined, the second part is reproduced by a symmetry operation. The thiophene ligand is disordered over two sets of positions with site-occupancy factors of 0.392(3) and 0.608(3). The hydrogen atoms were placed in calculated positions by means of a riding model with C–H = 0.95–0.99 Å and *Uk*_iso_ = 1.2*Uk*_eq_ (C). The crystal data parameters are summarized in Table S1.†

## 3 Experimental details

The UV-vis absorption measurements were carried out on a PerkinElmer Lambda800 instrument on an approx. 230 nm thick film prepared from a 20 mg mL^−1^*trans*-[Ru(dppe)_2_(T)_2_] chloroform solution. The glass substrate was sonicated in acetone and isopropanol bath for 10 minutes each. The solution was spin-coated on the glass substrate with a spin-rate of 500 rpm and an acceleration of 100 rpm s^−1^ for 60 s.

The thin films for photoemission spectroscopy were deposited on sputter cleaned gold and silver foils by powder sublimation in ultra high vacuum conditions (*p* = ≈10^−9^ mbar) with a growth rate of approximately 0.3 nm min^−1^, which was monitored by a quartz crystal micro balance. The strong acceptor F_6_TCNNQ, purchased from Novaled GmbH was used to form a charge transfer interface. The evaporation temperatures were approx. 270 °C for *trans*-[Ru(dppe)_2_(T)_2_] and 245 °C for F_6_TCNNQ. A monochromated Al-K_α_ X-ray source (*hν* = 1486.7 eV) was used for X-ray photoemission spectroscopy (XPS) and a non-monochromated UVS-300 (*hν* = 21.21 eV) He gas-discharge lamp for UV photoemission spectroscopy (UPS). The measurements were carried out with a SPECS PHOIBOS-150 analyser operating with pass energies of 3 eV for UPS and 10 eV for XPS. The XPS core level spectra were calibrated using the Au 4f_7/2_ and Ag 3d_5/2_ core level emission at a binding energy of 84.0 eV and 368.3 eV, and the UPS valence spectra are referred to the Fermi-cutoff of the metal substrates at 0.0 eV. Furthermore, the valence spectra were corrected for contributions of He1_β_ and He1_γ_ radiation. It was assumed that the spectra resulting from these satellites have the same shape with intensities of 1.8% (He1_β_) and 0.5% (He1_γ_) of the He1_α_ signals and are shifted by 1.87 eV (He1_β_) and 2.52 eV (He1_γ_). To obtain the correct high binding energy cutoff (HBEC), a bias voltage of 5 V was applied to the sample.

In addition to the quartz micro balance, the film thickness was estimated by the intensity variation of the substrate metal core level peak and applying the method of Seah and Dench,^15^ assuming a homogeneous film growth. The spectra presented in this study are referred to the ionisation energy (*E*_I_) which is given by:

1 *E*_I_ = *E*_B_ + *ϕ*

with the binding energy *E*_B_ and the work function

2 *ϕ* = 21.21 eV − HBEC

For the charge transfer interface measurements, the bottom layer with a thickness of 3 nm to 5 nm is deposited first, followed by the stepwise deposition of the top layers. Both deposition sequences, with the acceptor F_6_TCNNQ as bottom layer and with *trans*-[Ru(dppe)_2_(T)_2_] as bottom layer were prepared. In order to probe changes in the electronic configuration, the core levels (C 1s, P 2p, S 2p, Ru 3d, N 1s, F 1s and Au 4f) as well as the valence region and the HBEC are measured after each deposition step.

## 4 Spectroscopic analysis

### 4.1 Properties of thin films

**Fig. 3:**
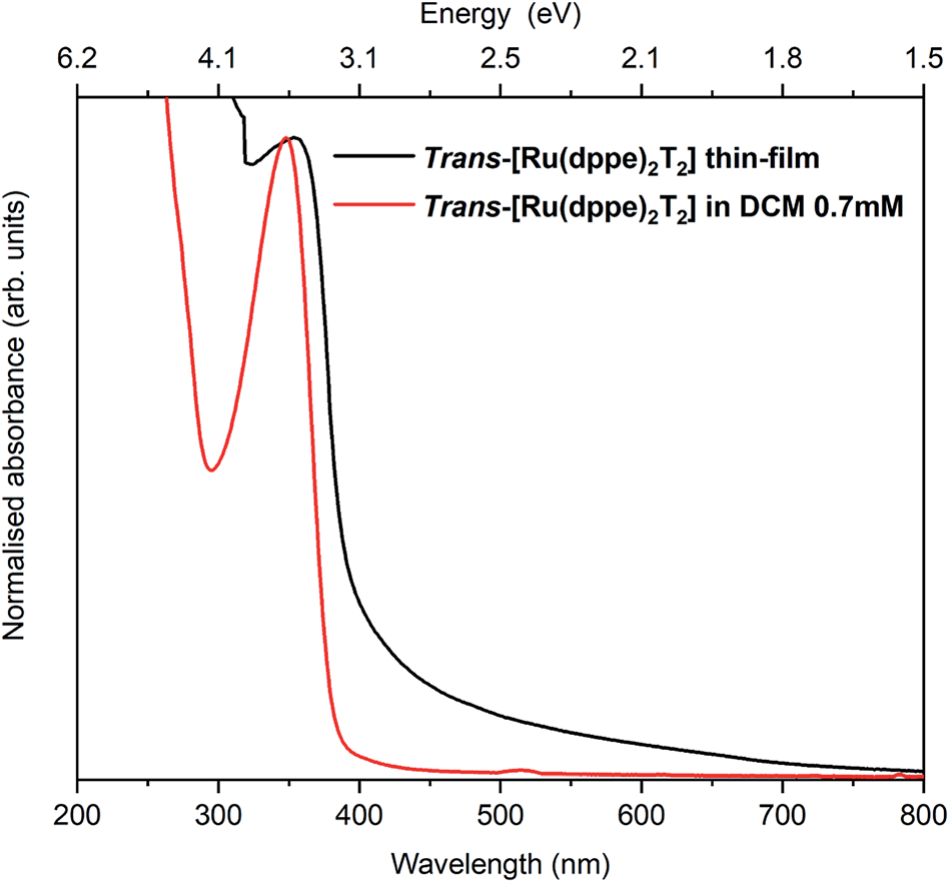
Absorption spectra of a spin coated RuC film on a glass substrate (black) and a DCM solution of 0.7 mmol L^−1^ RuC.

The electronic and optical properties of *trans*-[Ru(dppe)_2_(T)_2_] were studied in various ways. In the following, *trans*-[Ru(dppe)_2_(T)_2_] will be referred to as ruthenium complex (RuC). First, the optical absorption was measured in solution and solid form. The solution was prepared by dissolving the complex in dichloromethane with a concentration of 0.7 mmol L^−1^ and the film was prepared by spin-coating. Fig. 3 shows the absorption spectra of film and solution. The optical gap is determined as 3.1 eV for the film and 3.2 eV for the solution. The very similar values indicate that the intermolecular interactions in the film do not significantly differ from those of the molecules in solution.

**Fig. 4:**
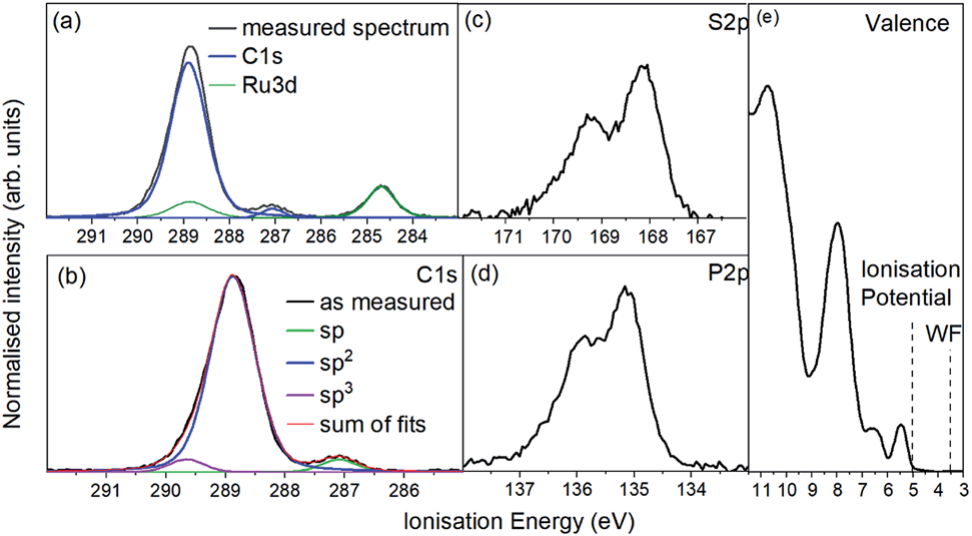
Photoemission spectra of a 7.0 nm RuC film on a silver foil. (a) C 1s (blue) and Ru 3d (green), (b) only C 1s line after subtraction of Ru 3d doublet, (c) S 2p, (d) P 2p and (d) UP valence spectrum.

For the photoemission study, a film of approx. 7.5 nm thickness was evaporated onto a silver foil. The XP spectra of the core levels as well as the UP spectra of the valence region are displayed in Fig. 4a shows the C 1s and Ru 3d region. It consists of one strong broad peak at an ionisation energy (*E*_i_) of about 288.8 eV with a smaller peak at 287.0 eV. The Ru 3d_5/2_ feature is detected at 284.7 eV with the 3d_3/2_ line superimposed by the broad C 1s peak. Fig. 4(b) shows the same spectrum after subtracting the Ru 3d features. The molecule contains 64 carbon atoms from which 56 are sp^2^ hybridised (double bonds) and 4 each sp (triple bonds) and sp^3^ hybridised (single bonds) giving a ratio of 14 : 1 : 1. We assign the feature at around 287 eV to the sp hybridised carbon atoms because the two electrons forming π-bonds provide additional screening of the C 1s photohole resulting in a lower binding energy and therefore a lower *E*_i_. Consequently the C 1s line of the sp^3^ hybridised carbons must lie at a higher *E*_i_ and the sp^2^ hybridised in between. The ratio of 14 : 1 : 1 could be reproduced by the area ratios of the fits presented in Fig. 4b.

The emission from S 2p and P 2p core levels (Fig. 4b and c) results in a broadened spin–orbit feature which is in agreement with the molecular structure and the equivalent positions of the S and P atoms. We assign the broadening to disorder in the films with respect to the molecular arrangement (Fig. 4b and c).

A comparison of the peak areas of the Ru 2p doublet and the C 1s peaks weighted with the atomic sensitivity factors and the number of respective atoms per molecule showed the same stoichiometric composition in the film as in the molecule, indicating that the molecules stayed intact during evaporation. Additionally, an IR spectrum of a film was compared to a powder spectrum which showed good agreement (ESI†).

**Fig. 5:**
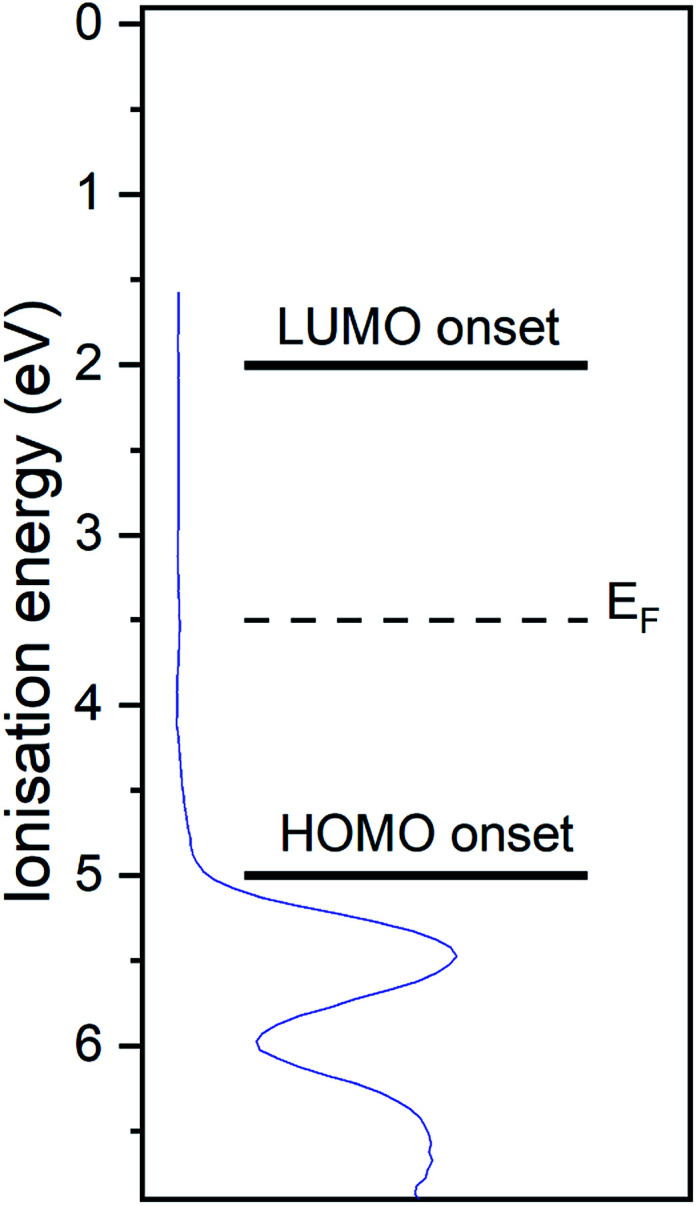
Energy level diagram of RuC. Values are based on photoemission and UV-vis spectra. Valence UP spectrum of 7.0 nm RuC on silver foil is shown in blue.

The UP spectrum of the valence region (Fig. 4d) shows the lowest ionisation energy feature of the film, representing the HOMO, with a spectral onset at 5.0 eV. This value was also found in cyclic voltammetry, which again confirms the similar intermolecular interaction in film and solution. The work function of the film, determined by the position of the HBEC, was 3.5 eV. Together with the optical gap of around 3 eV it can be concluded that the RuC is an organic intrinsic semiconductor with the Fermi energy in the middle of the HOMO–LUMO gap. An energy level diagram is shown in Fig. 5.

In order to study the charge transfer properties of the molecule, the strong electron acceptor hexafluorotetracyanonaphthoquinodimethane (F_6_TCNNQ)^16^ was used. Its reported electron affinity of 5.6 eV (ref. 17) is larger than the determined ionisation potential of 5.0 eV of the RuC, which should energetically enable a charge transfer from the RuC to F_6_TCNNQ. In the next section, the charge transfer reaction is analysed spectroscopically, first optically in a mixed solution and than at an interface with photoemission spectroscopy.

### 4.2 Charge transfer with acceptor F_6_TCNNQ

**Fig. 6:**
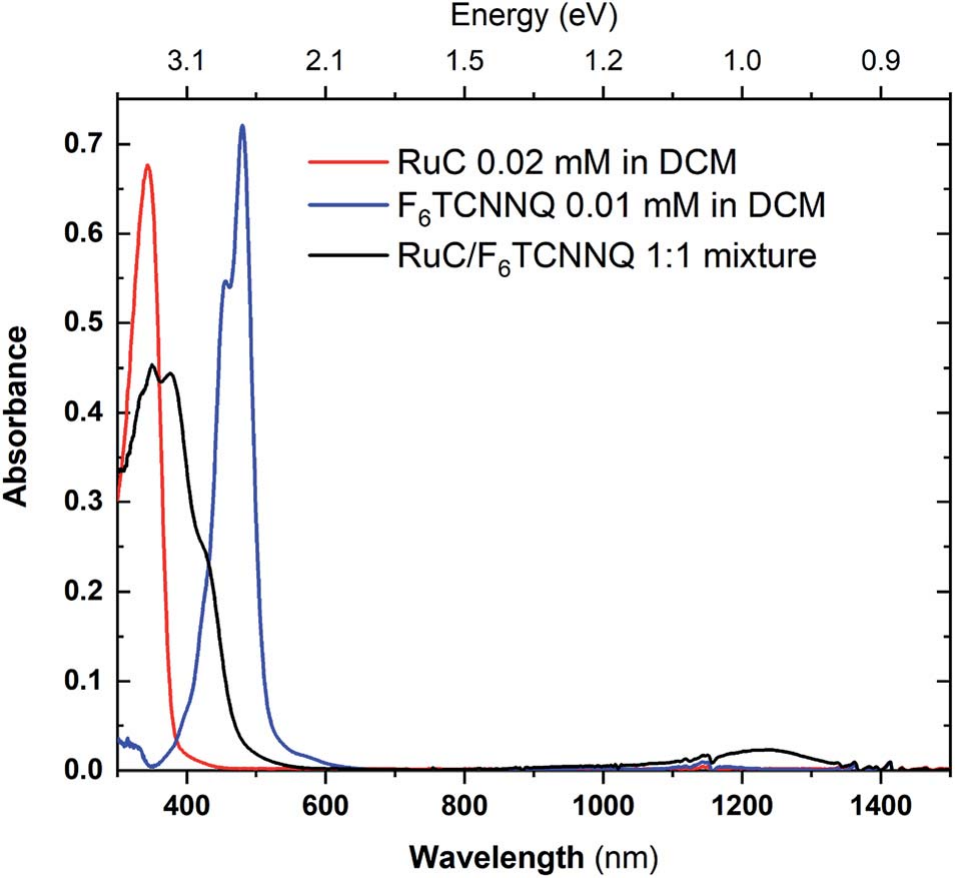
UV-vis absorption spectra of pristine materials RuC (red) and F_6_TCNNQ (blue) and the 1 : 1 mixture (black) solved in DCM.

In order to investigate a possible charge transfer reaction with the acceptor F_6_TCNNQ, both materials were mixed in a 1 : 1 ratio in solution. The UV-vis absorption spectra of the mixture as well as the pristine materials are shown in Fig. 6. The absorption spectrum of pristine F_6_TCNNQ is characterised by an excitation onset at around 500 nm which corresponds to an optical gap of around 2.4 eV which is in accordance to published values.^16^ The RuC optical absorption spectrum shows a strong peak with a spectral onset around 400 nm or 3.1 eV. Upon mixing of both materials a new broad feature below the optical gap of the pristine compounds appears at around 1250 nm. This peak is assigned to intermolecular charge transfer from the RuC to F_6_TCNNQ. Previous studies of molecular semiconductors doped with F_6_TCNNQ in solid and dissolved form have identified the absorption spectrum of reduced F_6_TCNNQ.^18–20^ According to those publications the spectrum of F_6_TCNNQ^−^ consists of a peak at 1140 nm which is followed by vibronic satellites at smaller wavelengths. The excitation energy in our case is smaller (around 960 nm) and we cannot resolve satellites. This indicates that no pure reduced F_6_TCNNQ is formed and instead some reaction between the two compounds is occurring. To further elucidate the charge transfer reaction, an interface of both material is investigated by photoemission spectroscopy.

The photoemission results of the charge transfer interface are displayed in the following manner: the sequence with the acceptor F_6_TCNNQ as bottom layer is shown on the left and the reverse direction on the right. The spectra are normalized and stacked in dependence of the film thickness in order to compare peak position and shape.

**Fig. 7:**
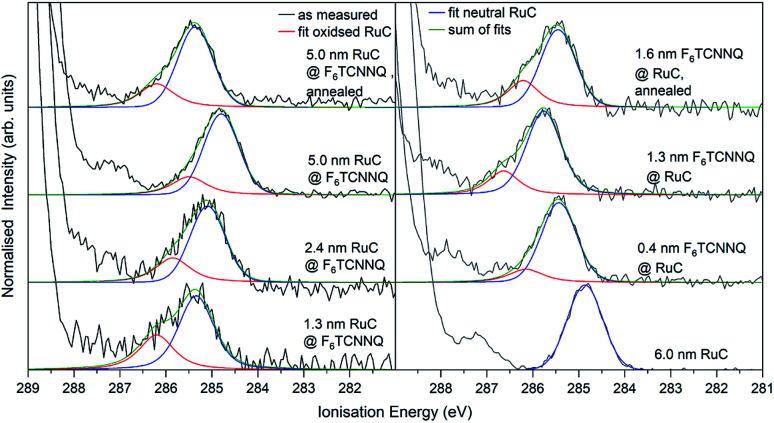
Ru 3d core level XPS spectra of RuC–F_6_TCNNQ interfaces; left: F_6_TCNNQ as bottom layer with stepwise deposition of RuC; right: opposite sequence. The fit obtained from the neutral spectrum (bottom right) is shown in blue. All other spectra can be reconstructed by a superposition of the neutral spectrum and a second contribution which is assigned to oxidised molecules (red). Spectra were fitted with PseudoVoigt profiles with the software Fityk.^25^

The XPS spectra of the Ru 3d_5/2_ core level spectra are shown in Fig. 7. The Ru 3d_3/2_ peak at 290.7 eV (ref. 21) is superimposed by the large C 1s peak that starts at an *E*_i_ of about 287 eV. The bottom curve on the right shows the Ru 3d_5/2_ feature of a pristine RuC film of 5.0 nm thickness that can be fitted by a Voigt profile with a predominantly Gaussian contribution. Deposition of a thin layer of F_6_TCNNQ (spectrum above) causes a shift of about 0.6 eV to higher *E*_i_. This is a first indication of a charge transfer from the RuC to F_6_TCNNQ since an accumulation of charge in the acceptor layer causes a dipolar layer at the interface which increases the energy required to emit a Ru 3d core level electron. The opposite energetic shift is observed for the opposite deposition sequence (left).

Apart from the energy shifts, changes in the spectral shape are observed. When evaporating a thin layer of RuC on F_6_TCNNQ (bottom left) a shoulder at higher *E*_i_ appears. Considering a charge transfer from the RuC to F_6_TCNNQ, the absence of a valence electron results in diminished screening of the photohole which leads to a higher ionisation energy for the core level electrons. Therefore the additional feature can be attributed to an oxidised Ru centre of the molecules. For higher coverages, the contribution of the RuC cation to the overall spectrum decreases because additional RuC molecules far from the interface region are not oxidised. The topmost spectrum was taken after tempering the sample with a 5.0 nm RuC coverage at 100 °C for 1 h. This lead to a mixing of the molecules by diffusion, resulting in a stronger cation signal. The opposite deposition sequence with the RuC as bottom layer and a stepwise deposition of F_6_TCNNQ is shown on the right. Here, the share of the cation signal is increasing for increasing F_6_TCNNQ coverage. This can be explained by the information depth of the photoelectron spectroscopy technique. With low F_6_TCNNQ coverage, the Ru 3d signal mainly stems from unreacted molecules far below the interface. With higher coverages, the spectrum is increasingly dominated by oxidised RuC molecules close to the interface, because electrons from deeper lying molecules cannot escape the sample anymore. Analysis of the S 2p and P 2p spectra revealed no change in peak shape in the interface region with F_6_TCNNQ. This indicates that predominantly the Ru centre of the RuC is oxidised while the ligand remains almost unaffected.

**Fig. 8:**
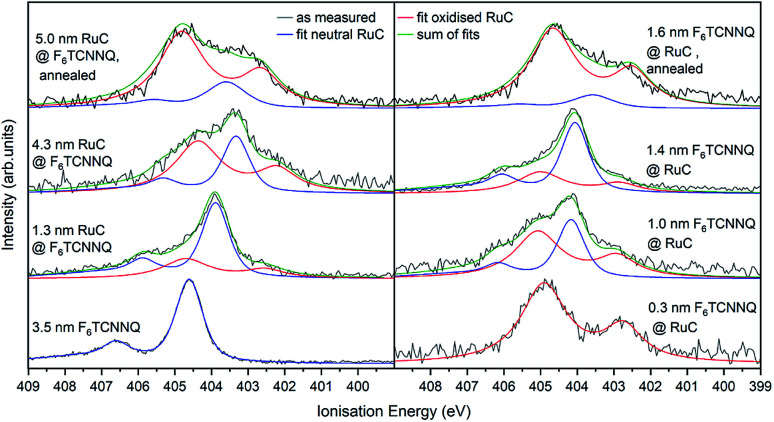
N 1s XPS spectra of RuC–F_6_TCNNQ interfaces; left: F_6_TCNNQ as bottom layer with stepwise deposition of RuC; right: opposite sequence. The neutral spectrum (bottom right) and the reduced spectrum (bottom left) were fitted by PseudoVoigt profiles (blue and red) curves. All other spectra could be reconstructed by a superposition of those two contributions.

In order to confirm the charge transfer, the N 1s core level of F_6_TCNNQ is analysed in Fig. 8. For pristine F_6_TCNNQ (bottom curve on the left), the spectrum consists of a main feature at an *E*_i_ of 405.6 eV and a satellite at 406.6 eV stemming from a π–π* shake-up process.^22^ Since the Ru-complex does not contain nitrogen, the bottom curve on the right shows already the 0.3 nm coverage of F_6_TCNNQ. The spectrum looks a lot different than the neutral spectrum with a large feature at 404.8 eV and a smaller one at 402.7 eV. Since a film thickness of 0.3 nm constitutes the sub-monolayer regime (assuming flat lying molecules) it can be assumed that all molecules are reduced. Hence, it should be possible to reconstruct all other spectra by superimposing the neutral spectrum (blue curves) and the anion spectrum (red curves). This is indeed possible. For the sequence with F_6_TCNNQ as bottom layer, the share of the anion spectrum to the total signal increases for growing film thicknesses. Analogous to the Ru 3d spectra, this can be explained by the surface sensitivity of the method, shifting the ‘information window’ towards the interface region with increasing film thickness. For the opposite sequence, the share of the anion contribution decreases with higher coverages because molecules deposited on top of the first couple of layers are not reduced anymore. Again, annealing the sample drastically increases the amount of F_6_TCNNQ molecules that find a ‘partner’ to interchange charge with, leading to a higher share of the anion spectrum to the overall N 1s signal for both deposition sequences.

Previous studies on F_6_TCNNQ deposited on gold,^23^ graphene^24^ and pentacene^9^ found that the N 1s anion spectrum was shifted in binding energy but did not differ significantly in peak shape from the neutral spectrum. In the present case the N 1s anion spectrum indeed differs strongly in peak shape. This indicates that in our case the nitrogen atoms are not equivalent anymore due to an asymmetric charge distribution on the molecule. This indicates a reaction of one of the amino groups of F_6_TCNNQ with the RuC. This result is in accordance with the optical absorption measurements (Fig. 6) which also indicate that the charge transfer is not purely ionic.

**Fig. 9:**
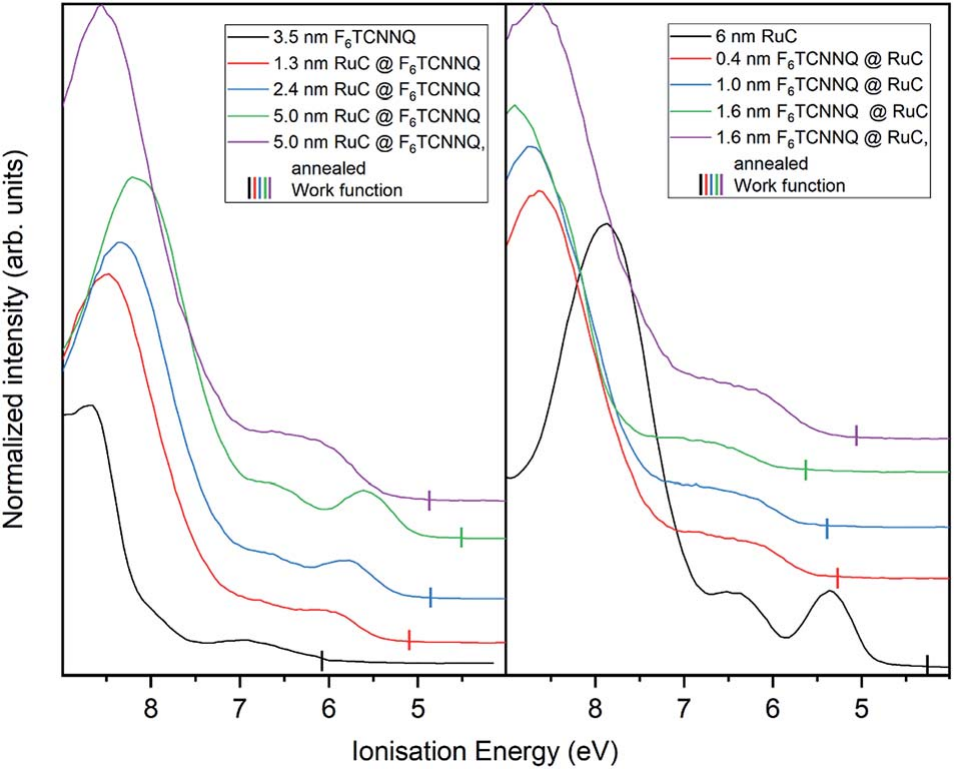
UP spectra of valence region of RuC–F_6_TCNNQ interfaces; left: F_6_TCNNQ as bottom layer with stepwise deposition of RuC; right: opposite sequence. The measured work functions for each deposition step are indicated by vertical lines.

Finally, the UP spectrum of the valence region is shown in Fig. 9 with the work function marked with vertical lines and again stacked in dependence of film coverage. The valence spectrum of pristine F_6_TCNNQ is shown on the left (black curve). The high intensity feature with an onset at around 8.0 eV can be attributed to the highest occupied molecular orbital (HOMO) of the molecule. The detected intensity at lower *E*_i_'s can be attributed to the gold substrate. Moreover, since the work function of gold (around 5 eV) is smaller than the electron affinity of F_6_TCNNQ (5.6 eV (ref. 17)), a charge transfer from the substrate to F_6_TCNNQ is possible. In this case, the intensity could be assigned to the filled former LUMO of the molecules in the interface region to the substrate.^23^

With increasing coverage of RuC, the previously discussed valence structure of the RuC appears. It is not possible to clearly identify the LUMO occupation of F_6_TCNNQ, since it is overlapping with the HOMO of the RuC. The reverse sequence shows the pristine RuC spectrum as bottom layer (black curve). With a low coverage of 0.4 nm of F_6_TCNNQ, the two low *E*_I_ features are not clearly distinguishable anymore, partly due to the attenuation of the RuC signal by F_6_TCNNQ and also the depletion of the RuC's HOMO and the filling of the overlapping F_6_TCNNQ LUMO. The two topmost violet curves show the spectra after tempering. Both spectra look similar with a broad feature with an onset of 5.5 eV. This is in consistence with the XPS results and shows intermixing of the molecules independent from the deposition sequence.

The work function (vertical lines in Fig. 9) increases by about 1 eV when depositing 0.4 nm of F_6_TCNNQ (Fig. 9 – right) while it decreases by roughly the same amount for the opposite deposition sequence (Fig. 9 – left). This is further indication of a charge transfer since it reflects an immediate shift of the Fermi level when the RuC is in contact with F_6_TCNNQ. Moreover, no occupied states close to the Fermi level (work function) are detected which indicates that the transferred charges are localised on their respective molecules.

## 5 Conclusion

In summary, we have synthesised an archetype ruthenium complex *trans*-[Ru(dppe)_2_(T)_2_] and determined its fundamental electronic and optical properties. Optical absorption spectroscopy on spin-coated thin films revealed an optical gap of 3.1 eV while an ionisation potential of about 5.0 eV was determined by photoemission spectroscopy on vacuum evaporated films. Electrochemical analysis yielded similar values for the molecule in solution. A charge transfer reaction with the strong electron acceptor F_6_TCNNQ was probed by optical absorption in a dissolved mixture of both materials. Furthermore, a charge transfer interface was formed by thermal evaporation and analysed by photoemission spectroscopy. Oxidised *trans*-[Ru(dppe)_2_(T)_2_] and reduced F_6_TCNNQ molecules were identified at the interface. The core level XPS spectra revealed that oxidation of *trans*-[Ru(dppe)_2_(T)_2_] occurs predominantly at the Ru centre while strong changes in the N 1s spectra of the reduced acceptor show that electron and hole are clearly separated. However, the XPS and UV-vis aborption spectra differ from those previously reported for pure anionic F_6_TCNNQ, which indicates that in the present case the charge transfer is not completely ionic.

## Conflicts of interest

There are no conflicts to declare.

## Supplementary Material

RA-010-D0RA08390A-s001

RA-010-D0RA08390A-s002
