# Supplementary material for: Synthesis and charge transfer characteristics of a ruthenium–acetylide complex
Source: RSC Adv. 2020 Nov 27;10(70):43242–7. doi: 10.1039/d0ra08390a (PMC9058256; doi:10.1039/d0ra08390a)
Supplement: RA-010-D0RA08390A-s001 [file RA-010-D0RA08390A-s001.pdf]

Electronic Supplementary Material (ESI) for RSC Advances.
This journal is © The Royal Society of Chemistry 2020

Supporting information

Instruments

Nuclear magnetic resonance (NMR)

NMR spectra were recorded on a Bruker Avance III 500 spectrometer operating at 500.13 MHz for 1H, 125.77 MHz for 13C, 470.59 MHz for 19F and 202.46 MHz for 31P using the standard pulse sequences included in the TOPSPIN 3.2 software package. The ^{1}H and ^{13}C NMR spectra were referenced on the solvent signal ($CDCl_3$: δ(1H) = 7.26 ppm, δ(13C) = 77.0 ppm; CD_2Cl_2: δ(^{1}H) = 5.31 ppm, δ(^{13}C) = 53.7 ppm). The ^{19}F NMR spectrum was referenced on external C_6F_6 (δ(^{19}F) = -163.0 ppm). All chemical shifts are reported in ppm.

Electrospray ionization mass spectrometry (ESI-MS)

Mass spectra were recorded on an Agilent 6200 Series Accurate-Mass Time-of-Flight (TOF) unit, equipped with a dual-nebulizer ion source connected to the 1 mL sample loop. After loading the sample into to sample loop, a steady solvent flow through the loop was obtained via manual attachment of the solvent outlet of a HPLC unit (Agilent 1100 Series unit (Agilent Technologies, Santa Clara, USA) equipped with degasser and a binary pump, set to 95 % Acetonitrile/5 % UHP water with 0.1 % formic acid and a flow rate of 0.5 mL/min). The ESI unit was operated in 4 GHz positive ion mode with nitrogen gas flow of 8 L/min at 325 °C, capillary voltage of 3500 V and fragmentor voltage of 175 V. For accurate mass determinations, spectra are calibrated with internal calibration standard/reference solution by use of a second reference sprayer orthogonal to the sample sprayer in the ion source. Mass calibration was performed with an Agilent tune mix from 100 to 2200 m/z prior measurement.

Reagents and Supplies. All organic solvents for analytical measurements were purchased from IRIS Biotech GmbH (Marktredwitz, Germany). THF was purchased from Sigma-Aldrich (Darmstadt, Germany). Ultra-high purity (UHP) water was prepared to 18.2 MΩ cm at 25˚C on a Milli-Q® Integral System (Merck Millipore, Darmstadt, Germany). All reagents were used without preliminary purification.

Sample Preparation. Samples were dissolved in 100 % THF to a concentration of 0.001 µmol/µL. 100 µl of the sample solution was loaded into a 1 mL sample loop filled with 100 % THF and 400 µl THF were added to the sample loop after loading the sample solution. The samples were directly injected into the mass spectrometer unit without purity determination.

Cyclic voltammetry

CV was measured on IVIUM under N_2 flow with an in-house three electrode system consisting a Pt disc working electrode (WE), Pt wire counter electrode (CE) and Ag/Ag+ reference electrode (RE). During CV measurements, 0.1M of n-Bu4NPF6 in acetonitrile was used as supporting electrolyte and ferrocene was used as the internal reference. The scan rate was 50mV/s.

Raman Spectroscopy

Raman spectroscopy was obtained on RAMAN imaging system WITEC alpha300R with the excitation laser at 532nm and 0.005mW laser power.

X-ray crystallography

X-ray single-crystal data were collected at 160 [1] K on a XtaLAB Synergy Dualflex (Pilatus 200K detector) diffractometer[1] with an Oxford liquid-nitrogen Cryostream cooler. The crystal data collection and structure refinement parameters are summarized in Table S1.

IR Spectroscopy

IR Spectroscopy was performed at room temperature and atmospheric pressure using a Bruker Vertex spectrometer equipped with a microscope.

Synthetic procedures

Synthesis of *cis*-$Ru(dppe)_2Cl_2$ (5)

Modified after a literature report[1]

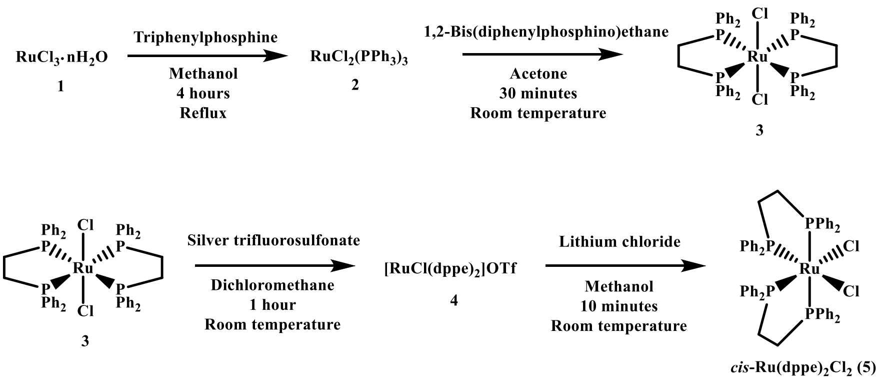

Tris(triphenylphosphine)ruthenium(II) chloride (2) 100 ml of analytic grade methanol was added into a 250 ml 1-neck-round-bottom flask. Triphenylphosphine (12 g, 45.82 mmol) was added into the solvent, followed by the addition of ruthenium trichloride hydrate **(1)** (2 g, 7.66 mmol), resulting in a deep brown color solution. The mixture was heated to reflux for 4 hours. The mixture was then filtered, washed with diethyl ether and dried in air. Tris(triphenylphosphine)ruthenium(II) chloride **(2)** was obtained (6.4 g, 6.65 mmol, 86.9%). ^{31}**P NMR** (202.46 MHz, $CDCl_3$) δ: 28.94 (s).

***Trans*-$Ru(dppe)_2Cl_2$ (3)** Tris(triphenylphosphine)ruthenium(II) chloride **(2)** (6.4 g, 6.65 mmol) was dissolved into 70 ml of reagent grade, giving a deep brown color solution. 1,2-Bis(diphenylphosphino)ethane (5.6 g, 13.97 mmol) was then added into the solution. A yellow colored powder precipitated after 1 hour of stirring at room temperature. The mixture was filtered and the obtained yellow solid washed with acetone and dried in air. The resulting yellow powder was recrystallized in chloroform to afford *trans*-$Ru(dppe)_2Cl_2$ **(3)** in crystalline form (4.9 g, 5.09 mmol, 76.5%).^{31}**P NMR** (202.46 MHz, $CDCl_3$) δ: 45.42 (s).

$[RuCl(dppe)_2]OTf$ (4) *Trans*-$Ru(dppe)_2Cl_2$ **(3)** (4.9 g, 5.09 mmol) was dissolved in 75 ml dichloromethane, followed by the addition of silver trifluromethane-sulfonate (1.3 g 5.10 mmol), giving a deep red solution. The mixture was stirred at room temperature and after 1 hour, filtered over a pack of celite. The filtrate was collected, diluted with hexanes and concentrated at the rotary evaporator. The product **(4)** was collected as a dark red powder (4.7 g, 4.3 mmol, 84.6%). ^{31}**P NMR** (202.46 MHz, $CDCl_3$) δ: 84.04 (s), 54.87 (s). ^{19}**F NMR** (470.59 MHz, $CDCl_3$) δ: -79.06 (s).

***Cis*-$Ru(dppe)_2Cl_2$ (5)** $[RuCl(dppe)_2]OTf$ **(4)** (4.7 g, 4.3 mmol) was dissolved in 600 ml of methanol. Lithium chloride (2.2 g, 52.0 mmol, 12.1 eq.) was added into the solution. A yellow powder precipitated immediately after the addition. The mixture was stirred at room temperature for 10 minutes and the powder was collected and washed with methanol. It was air dried and a yellow powder **(5)** was obtained (3.8 g, 3.94 mmol, 91.5%. Overall yield = 53%). ^{31}**P NMR** (202.46 MHz, $CDCl_3$) δ: 52.37 (s), 37.84 (s).

<u>Synthesis of *Trans*-[Ru(dppe)$_2$T$_2$]</u>

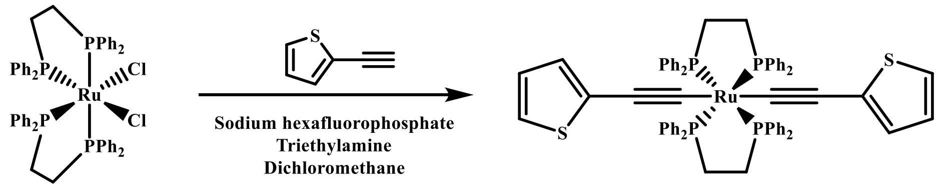

cis-Ru(dppe)$_2$Cl$_2$ (5) *Trans*-[Ru(dppe)$_2$T$_2$]

***Trans*-[Ru(dppe)$_2$T$_2$]** *Cis*-Ru(dppe)$_2$Cl$_2$ **(5)** (0.5 g, 0.52 mmol) was added into a 100 ml round-bottom flask with 50 ml of dichloromethane. Sodium hexafluorophosphate (0.29 g, 1.70 mmol, 3.3 eq.) was then added into the same flask. Once the hexafluorophosphate was added, the color of the solution changed from bright yellow to deep red immediately. 2-ethynylthiophene (0.18 g, 1.62 mmol, 3.1 eq.) and trimethylamine (0.32 g, 3.13 mmol, 6.02 eq.) were then added into the mixture. The color of the mixture turned from red to yellow. The reaction mixture was stirred at room temperature overnight, then, ACN was added into the resulting solution and the DCM was removed on rotary evaporator. The resulting precipitate was washed with ACN and then hexanes. The target compound was afforded as a yellow powder (0.49 g, 0.44 mmol, yield = 84.9%). **^{1}H NMR** (500.13 MHz, CD_2Cl_2) δ: 7.47 (m, 16H, phenyl-H), 7.20 (t, *J* = 7.4 Hz, 8H, phenyl-H), 7.02 (t, *J* = 7.6 Hz, 16H, phenyl-H), 6.83 (m, 4H, thienyl-H), 6.36 (t, *J* = 2.2 Hz, 2H, thienyl-H), 2.63 (m, 8H, alkyl-H on dppe). **^{13}C NMR** (125 MHz, CD_2Cl_2) δ: 138.31, 137.52, 134.71, 132.53, 129.29, 127.73, 126.63, 124.99, 20.24, 109.31, 31.97. **^{31}P NMR** (202.46 MHz, CD_2Cl_2) δ: 52.88 (s). **ESI MS** m/z: 1112.1662 gmol^{-1}. **Raman** (532 nm, cm^{-1}): 2064 cm^{-1} ($v_{C\equiv C}$). **Anal. Calcd.** For $C_{64}H_{54}P_4RuS_2$: C: 69.11; H: 4.89; S: 5.77. Found: C: 64.08; H: 4.42; S: 5.37.

NMR

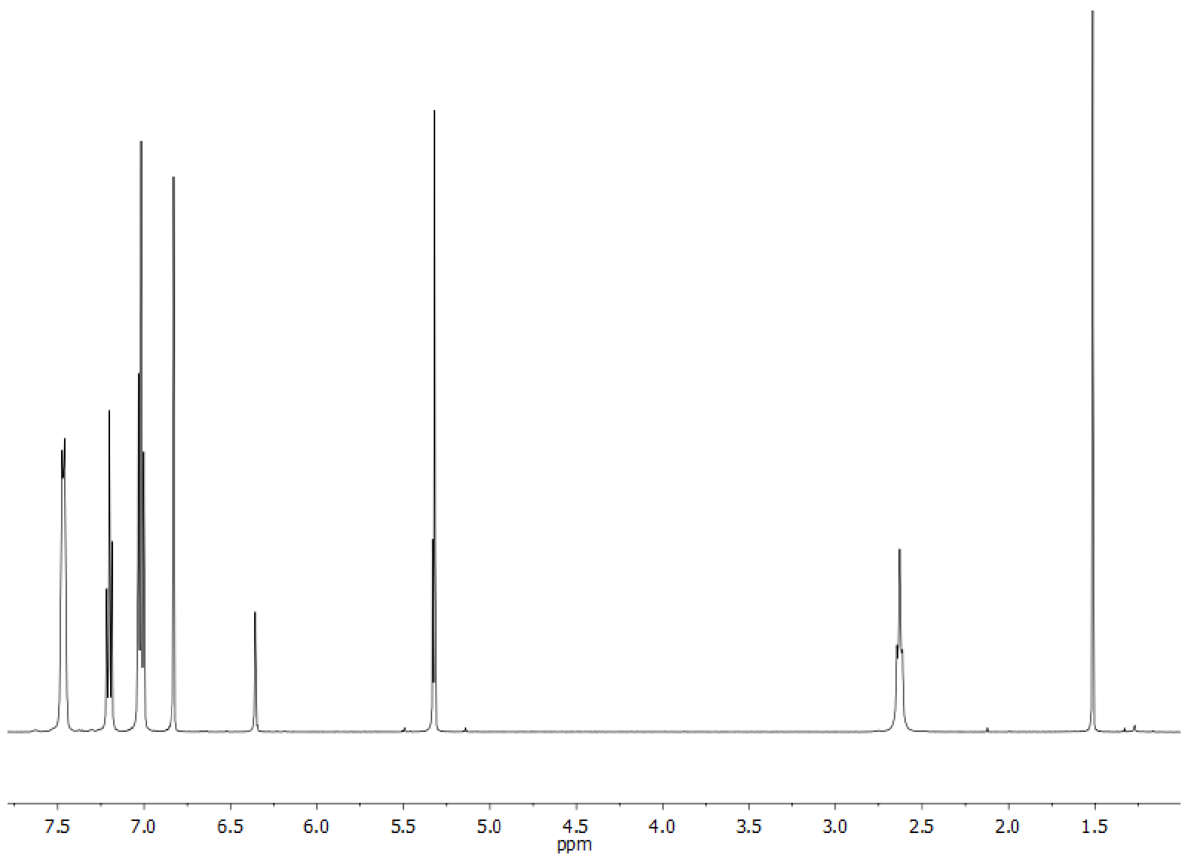

Figure S1: ^1H NMR of *trans*-[Ru(dppe)$_2$T$_2$] in CD_2Cl_2 (assignments see above)

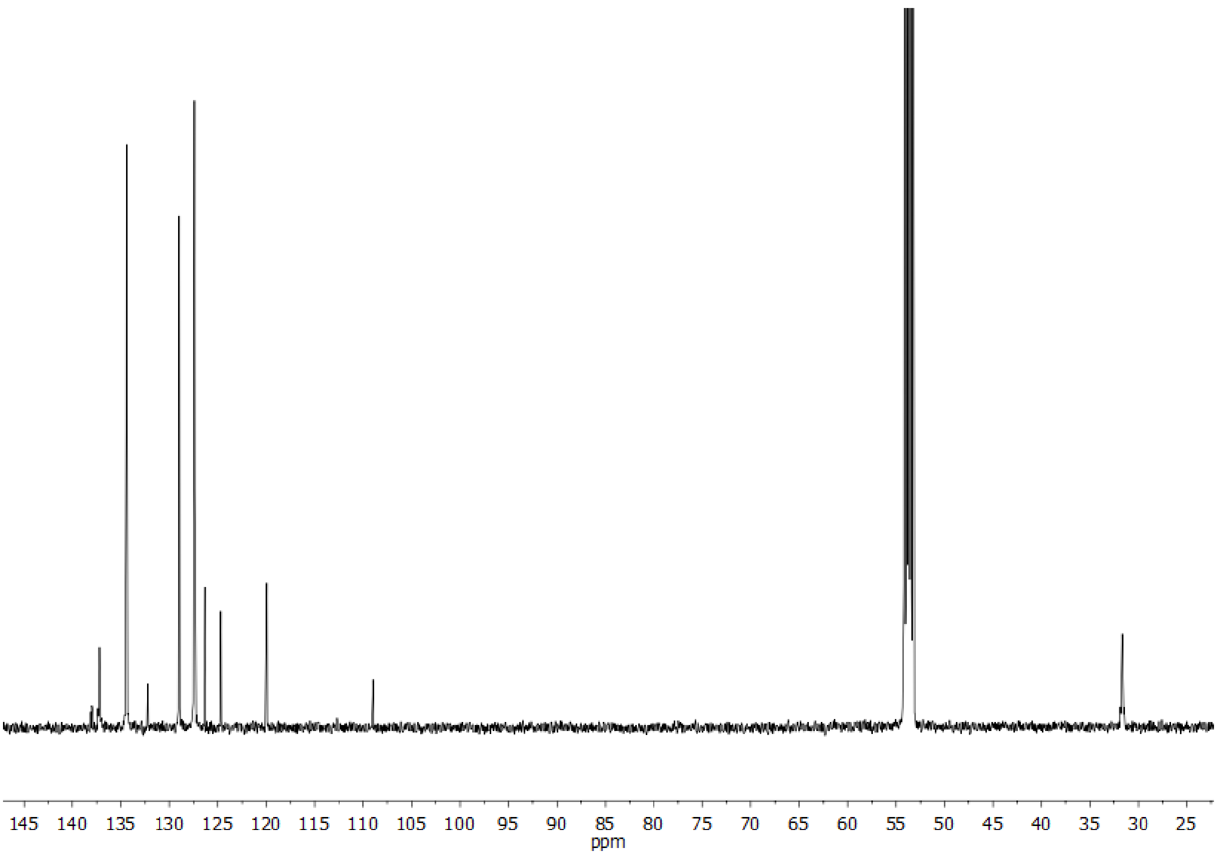

Figure S2: ^{13}C NMR of *trans*-[Ru(dppe)$_2$T$_2$] in CD_2Cl_2 (assignments see above)

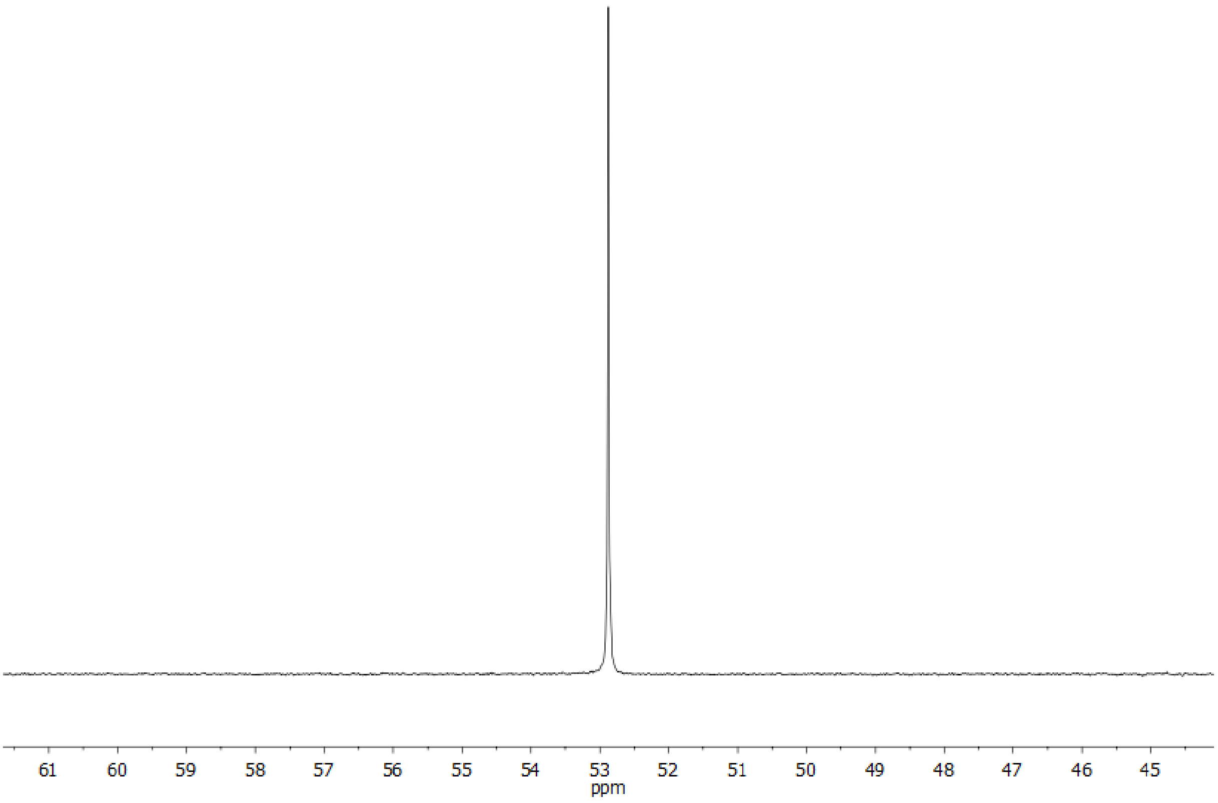

Figure S3: ^{31}P NMR of *trans*-[Ru(dppe)$_2$T$_2$] in CD_2Cl_2 (assignment see above)

Cyclic voltammetry

2.5 mg/ml of the Ru-complex in DCM was prepared and degassed prior to every measurement. The graphs below are already normalized against ferrocene/ferrocenium. In Figure S1, there is a fully reversible redox event at the range of -0.6 V to 0.6 V. The E_{pa} is -0.027 V and the E_{pc} is -0.12 V. Therefore, the $E_{1/2}$ is -0.073 V. The E_{onset} is at -0.14 V. Therefore, according to the following equation, the HOMO level is calculated to be -4.95 eV [2].

$$\mathrm{HOMO} = -\left(5.09 + \mathrm{E_{onset}} - \mathrm{E_{1/2,Fc/Fc+}}\right)\,\mathrm{eV}$$

Further oxidation result in an oxidation at 0.55 V, which is not fully reversible (Figure S2). No reduction is observed.

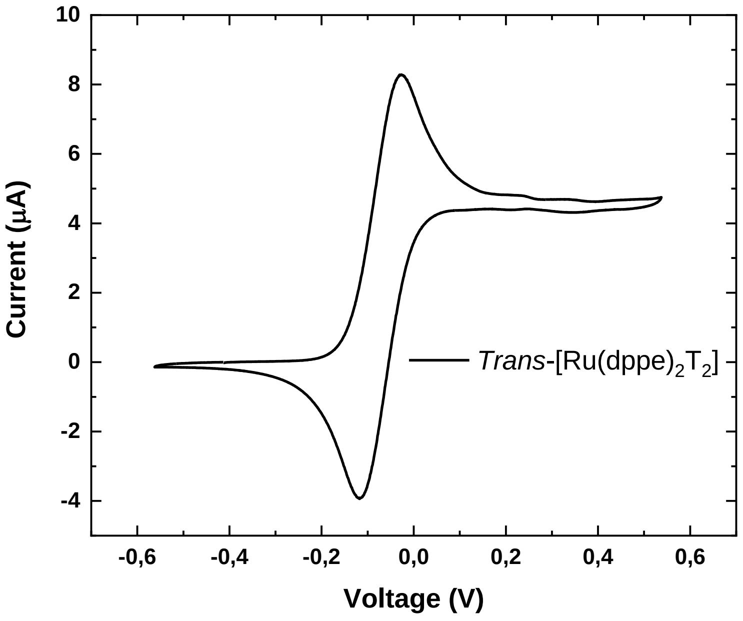

Figure S4: Cyclic voltammetry of *trans*-[Ru(dppe)$_2$T$_2$] between -0.6 V and 0.6 V

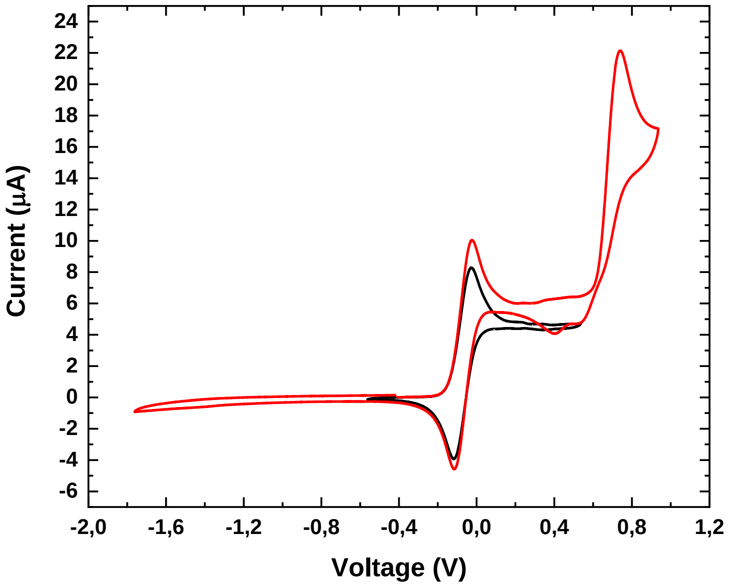

Figure S5: Cyclic voltammetry of *Trans*-[Ru(dppe)$_2$T$_2$] from -1.7 V to 1.1 V

Raman

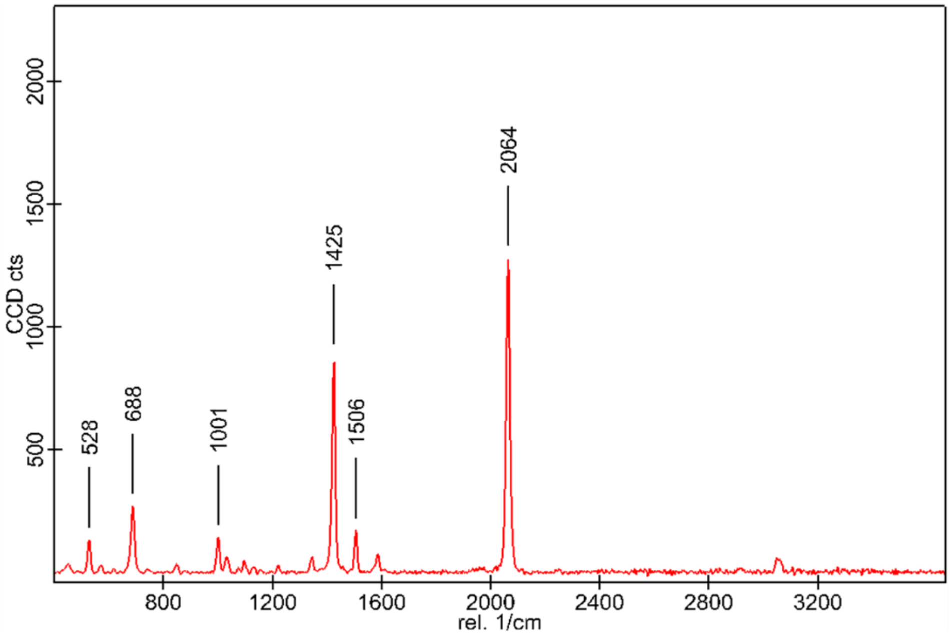

Figure S6: Raman spectrum of *trans*-[Ru(dppe)$_2$T$_2$] (400-3600 cm^{-1}), baseline corrected

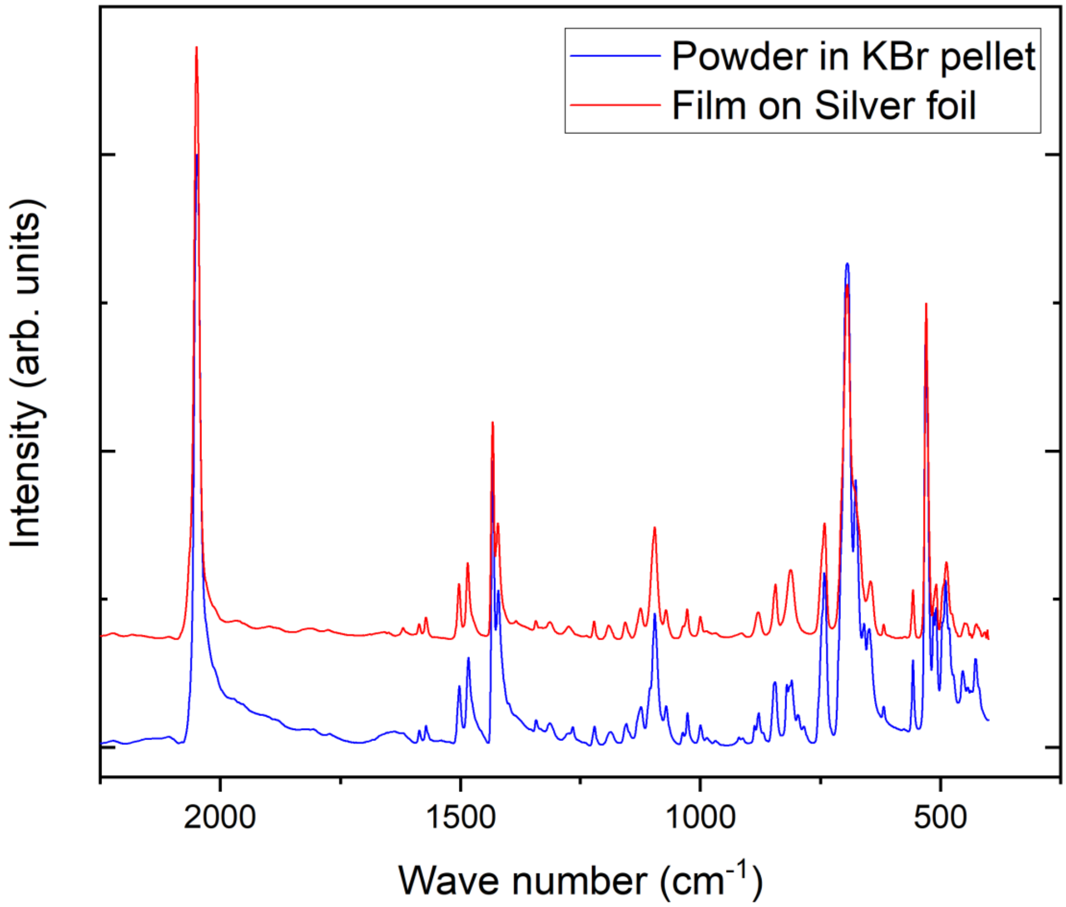

Figure S7: IR spectrum of *trans*-[Ru(dppe)$_2$T$_2$] as powder in KBr pellet and evaporated as thin film on silver foil.

X-ray crystallography

Table S1. Crystal data and structure refinement parameters for **Trans-[Ru(dppe)$_2$T$_2$]**.

	**Trans-[Ru(dppe)$_2$T$_2$]**
CCDC number	2010145
Empirical formula	$C_{64}H_{54}P_4RuS_2$
Formula weight	1112.14
Temperature/K	160(1)
Crystal system	triclinic
Space group	P-1
a/Å	9.46352(14)
b/Å	12.66738(18)
c/Å	12.98267(14)
α/°	116.7842(13)
β/°	100.5828(12)
γ/°	98.7407(12)
Volume/Å^3	1315.96(3)
Z	1
ρ_{calc}g/cm^3	1.403
μ/mm^{-1}	4.616
F(000)	574.0
Crystal size/mm^3	0.1 × 0.07 × 0.04
Radiation	CuKα (λ = 1.54184)
2Θ range for data collection/°	7.96 to 148.992
Index ranges	-10 ≤ h ≤ 11, -15 ≤ k ≤ 15, -16 ≤ l ≤ 15
Reflections collected	24930
Independent reflections	5369 [R_{int} = 0.0342, R_{sigma} = 0.0277]
Data/restraints/parameters	5369/76/341
Goodness-of-fit on F^2	1.049
Final R indexes [I>=2σ (I)]	R_1 = 0.0250, wR_2 = 0.0641
Final R indexes [all data]	R_1 = 0.0254, wR_2 = 0.0643
Largest diff. peak/hole / e Å^{-3}	0.62/-0.50

Literature

[1] M. A. Fox, J. E. Harris, S. Heider, V. Pérez-Gregorio, M. E. Zakrzewska, J. D. Farmer, D. S. Yufit, J. A. K. Howard and P. J. Low, *J. Organomet. Chem.* **2009**, 694, 2350–2358.

[2] Cardona, Claudia M., et al., *Advanced materials* 23.20 (2011): 2367-2371.
